# Immunotoxicity of silver nanoparticles in an intravenous 28-day repeated-dose toxicity study in rats

**DOI:** 10.1186/1743-8977-11-21

**Published:** 2014-05-07

**Authors:** Rob J Vandebriel, Elisa CM Tonk, Liset J de la Fonteyne-Blankestijn, Eric R Gremmer, Henny W Verharen, Leo T van der Ven, Henk van Loveren, Wim H de Jong

**Affiliations:** 1 Centre for Health Protection, National Institute for Public Health and the Environment, PO BOX 1, Bilthoven, BA 3720, The Netherlands

**Keywords:** Nanosilver, Repeated-dose toxicity, T-cell dependent antibody response, Keyhole limpet hemocyanin, Benchmark dose, Immunosuppression

## Abstract

**Background:**

Nanosilver is used in a variety of medical and consumer products because of its antibacterial activity. This wide application results in an increased human exposure. Knowledge on the systemic toxicity of nanosilver is, however, relatively scarce. In a previous study, the systemic toxicity of 20 nm silver nanoparticles (Ag-NP) was studied in a 28-day repeated-dose toxicity study in rats. Ag-NP were intravenously administered with a maximum dose of 6 mg/kg body weight (bw)/day. Several immune parameters were affected: reduced thymus weight, increased spleen weight and spleen cell number, a strongly reduced NK cell activity, and reduced IFN-γ production were observed.

**Methods:**

Prompted by these affected immune parameters, we wished to assess exposure effects on the functional immune system. Therefore, in the present study the T-cell dependent antibody response (TDAR) to keyhole limpet hemocyanin (KLH) was measured in a similar 28-day intravenous repeated-dose toxicity study. In addition, a range of immunological parameters was measured. Data obtained using the benchmark dose (BMD) approach were analyzed by fitting dose-response models to the parameters measured.

**Results:**

A reduction in KLH-specific IgG was seen, with a lowest 5% lower confidence bound of the BMD (BMDL) of 0.40 mg/kg bw/day. This suggests that Ag-NP induce suppression of the functional immune system. Other parameters sensitive to Ag-NP exposure were in line with our previous study: a reduced thymus weight with a BMDL of 0.76 mg/kg bw/day, and an increased spleen weight, spleen cell number, and spleen cell subsets, with BMDLs between 0.36 and 1.11 mg/kg bw/day. Because the effects on the spleen are not reflected by increased KLH-specific IgG, they, however, do not suggest immune stimulation.

**Conclusions:**

Intravenous Ag-NP administration in a 28-day repeated-dose toxicity study induces suppression of the functional immune system. This finding underscores the importance to study the TDAR to evaluate immunotoxicity and not to rely solely on measuring immune cell subsets.

## Background

Silver nanoparticles (Ag-NP) are frequently used in consumer and medical products because of their antimicrobial activity [1-5]. Despite the increased use of nanosilver-containing products [5-7], there is only limited information on the possible risks of exposure to Ag-NP.

Oral 28-day, oral 90-day, inhalation 28-day, and inhalation 13-week repeated-dose exposure studies did not show severe systemic toxicity of nanosilver [8-13]. This is probably because of the relatively low systemic exposure to nanosilver that is due to its low absorption from the gastro-intestinal tract (GI-tract) and lungs. However, clinical chemistry showed an increase in alkaline phosphatase (ALP), aspartate aminotransferase (ALT), and cholesterol, indicating liver toxicity [9,11,13]. Moreover, bile duct hyperplasia was seen [10,11]. We have recently performed a 28-day repeated-dose toxicity study [14] in which nanosilver was administered intravenously. This exposure route was chosen in order to avoid the limited systemic exposure due to the cellular barriers present in the GI-tract and lungs. Several immune parameters were affected: reduced thymus weight, increased spleen weight and spleen cell number, strongly reduced NK cell activity, and reduced IFN-γ production were observed.

A next step in the risk assessment of Ag-NP immunotoxicity is to evaluate exposure effects on a functional immune response. Therefore, in the present study the T-cell dependent antibody response (TDAR) to keyhole limpet hemocyanin (KLH) was determined in an intravenous 28-day repeated-dose toxicity study. In addition, a range of immunological parameters was measured, such as weights of organs of the immune system, hematology parameters, spleen cell subsets, and cytokine production.

## Results

### Effects of KLH immunization

KLH immunization did not affect body or organ weights. Immunization resulted in a 2% increase in mean corpuscular hemoglobin (MCH) (*P* = 0.007), a 4% decrease in hemoglobin distribution width (HDW) (*P* = 0.046), and a 17% increase in mean platelet volume (MPV) (*P* = 0.004). All other red blood cell and hemoglobin parameters were unaffected.

KLH immunization did not affect white blood cell parameters and did not affect bone marrow (BM) cellularity. Immunization did not affect spleen cell numbers, or percentages or numbers of the spleen cell populations analyzed (CD3, CD4, CD8, CD161a, and CD45RA).

KLH immunization resulted in a 30- to 60-fold increase in KLH-specific IgG compared to the non-specific background level (*P* < 0.001 and *P* = 0.009 for the control and 6 mg/kg Ag-NP groups, respectively). Immunization did not significantly increase KLH-specific IgM above the non-specific background level.

KLH immunization did not affect cytokine production.

### Effects of Ag-NP exposure

Table 1 shows for the various parameters evaluated the benchmark response (BMR), and the benchmark dose (BMD) with its 90% confidence interval (CI), the lowest 5% lower confidence bound (BMDL) and the highest 95% upper confidence bound (BMDU). Since the BMDL is considered the starting point for risk assessment [15], the presentation of the results is according to the BMDL.

**Table 1:** Summary of BMDs BMR, benchmark response; BMD, benchmark dose; CI, 90% confidence interval, with BMDL (lowest 5% lower confidence bound) and BMDU (highest 95% upper confidence bound) indicated; statistical model: 2 (E2/H2), 3 (E3/H3), and 5 (E5/H5)

**Parameter**	**BMR (5%)**	**BMD (mg/kg bw, day)**	**CI (5% - 95%)**	**Statistical model**
Body weight	**↓**	2.85	1.84 – 5.72	2
Spleen weight	**↑**	0.98	0.76 – 1.28	2
Thymus weight	**↓**	1.31	0.76 – 3.03	2
RBC	**↑**	2.64	2.04 – 3.62	2
RDW	**↑**	1.79	1.50 – 2.02	5
HDW	**↑**	1.89	1.28 – 2.48	5
MCV	**↓**	1.95	1.77 – 2.11	5
MCH	**↓**	1.76	1.39 – 1.91	5
MCHC	**↓**	7.26	5.65 – 10.2	2
% neutrophils	**↑**	1.23	0.69 – 3.76	2
% lymphocytes	**↓**	4.15	2.57 – 10.0	2
% monocytes	**↑**	0.69	0.48 – 1.01	2
% large unstained cells	**↑**	3.74	1.24 – 4.31	3
% reticulocytes	**↑**	1.23	0.85 – 1.96	2
# monocytes	**↑**	0.59	0.35 – 1.16	2
Spleen cell number	**↑**	1.03	0.74 – 1.51	2
% CD3	**↑**	1.41	1.09 – 1.88	2
% CD4	**↑**	1.47	1.11 – 2.04	2
% CD8	**↑**	1.11	0.73 – 1.95	2
% CD161a	**↑**	0.89	0.62 – 1.34	2
CD3/CD45RA ratio	**↑**	1.21	0.86 – 1.85	2
# CD3	**↑**	0.63	0.45 – 0.86	2
# CD4	**↑**	0.64	0.46 – 0.88	2
# CD8	**↑**	0.57	0.39 – 0.84	2
# CD161a	**↑**	0.52	0.36 – 0.72	2
# CD45RA	**↑**	1.10	0.68 – 2.23	2
KLH-IgG	**↓**	0.73	0.40 – 1.63	2
KLH-IgM	**↑**	5.28	3.64 – 6.01	3
Thymus IL-10	**↓**	1.17	0.58 – 4.53	2
Thymus IL-17	**↓**	2.79	1.47 – 6.60	2
Spleen IL-1β	**↑**	2.74	1.57 – 6.94	2
Spleen IL-6	**↑**	2.22	1.21 – 6.20	2

%, percentage; #, number

### Body and organ weights

Body weight was decreased by Ag-NP exposure, with a BMDL of 1.84 mg/kg bw/day, and thymus weight was decreased, with a BMDL of 0.76 mg/kg. Spleen weight was increased, with a BMDL of 0.76 mg/kg. There was no effect of Ag-NP exposure on liver and kidney weights. The original data are shown in Additional file 1: Table S1.

### Bone marrow cellularity

No exposure effect on bone marrow (BM) cellularity was seen. The original data are shown in Additional file 1: Table S2.

### Red blood cell and hemoglobin parameters

The number of red blood cells (RBC), red cell distribution width (RDW), and hemoglobin distribution width (HDW) were increased by Ag-NP exposure, with BMDLs of 2.04, 1.50, and 1.28 mg/kg, respectively. Mean corpuscular volume (MCV), mean corpuscular hemoglobin (MCH), and mean corpuscular hemoglobin concentration (MCHC) were decreased, with BMDLs of 1.77, 1.39, and 5.65 mg/kg, respectively. The other red blood cell and hemoglobin parameters evaluated; hemoglobin concentration (HGB), hematocrit (HCT), platelet count (PLT), and mean platelet volume (MPV) were unaffected by exposure. The original data are shown in Additional file 1: Table S2.

### White blood cell parameters

The percentages of neutrophils, monocytes, large unstained cells, and reticulocytes were increased by Ag-NP exposure, with BMDLs of 0.69, 0.48, 1.24, and 0.85 mg/kg, respectively. The number of monocytes was increased, with a BMDL of 0.35 mg/kg. The percentage of lymphocytes was decreased, with a BMDL of 2.57 mg/kg. The percentages of eosinophils and basophils were unaffected.

The numbers of neutrophils, lymphocytes, eosinophils, large unstained cells, basophils, and reticulocytes were unaffected, however, suggesting a minor effect of Ag-NP exposure on the distribution of white blood cells. The original data are shown in Additional file 1: Table S3.

### Spleen cell populations

Spleen cell number and percentages of CD3, CD4, and CD8 cells were increased by Ag-NP exposure, with BMDLs of 0.74, 1.09, 1.11, and 0.73 mg/kg, respectively. The numbers of CD3, CD4, and CD8 cells were increased, with BMDLs of 0.45, 0.46, and 0.39 mg/kg, respectively.

The percentage and number of CD161a cells was increased, with BMDLs of 0.62 and 0.36 mg/kg, respectively. The percentage of CD45RA cells was unaffected by exposure, while the number of CD45RA cells was increased, with a BMDL of 0.68 mg/kg. The CD3/CD45RA ratio was increased, with a BMDL of 0.86 mg/kg. The original data are shown in Additional file 1: Table S4.

### KLH-specific IgG and IgM

KLH-specific IgG was decreased by Ag-NP exposure, with a BMDL of 0.40 mg/kg. KLH-specific IgM was increased by Ag-NP exposure, with a BMDL of 3.64 mg/kg. The original data are shown in Additional file 1: Table S5.

### Cytokine production

IL-10 and IL-17 production by Con A-stimulated thymocytes were decreased by Ag-NP exposure, with a BMDL of 0.58 and 1.47 mg/kg, respectively. IL-1β and IL-6 production by Con A-stimulated splenocytes were decreased with a BMDL of 1.57 and 1.21 mg/kg, respectively. IFN-γ, IL-2, and TNF-α production by Con A-stimulated thymocytes, and IL-2 and IL-10 production by Con A-stimulated splenocytes were unaffected by exposure. The original data are shown in Additional file 1: Table S6.

### Comparison of BMD values

**Figure 1:**
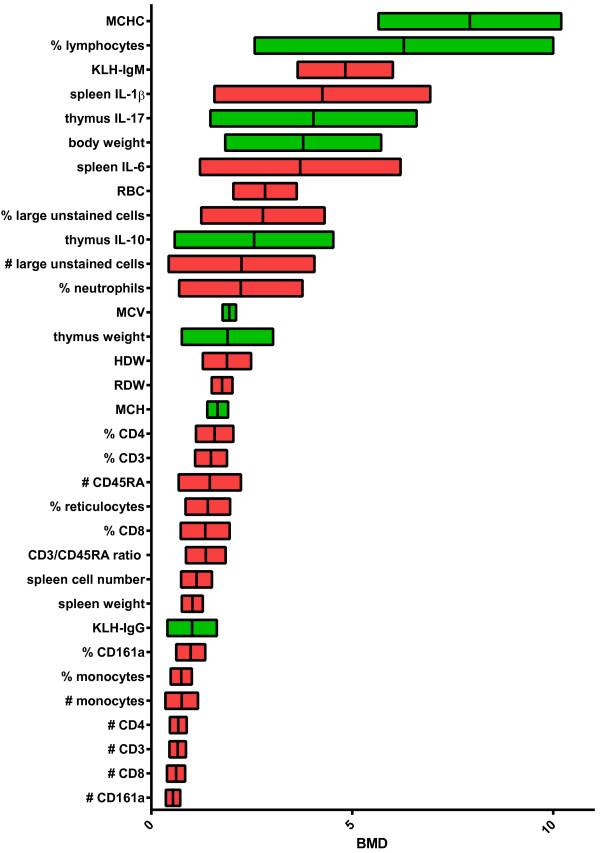
**BMD with 90% CI.** BMR = 5%. Red: increased; Green: decreased. Sorted by BMD.

In Figure 1, the parameters measured are sorted according to their BMDL. Among the most sensitive parameters are the increased numbers of spleen monocytes, NK cells (CD161a), and T-cells (CD3, CD4, and CD8). The increased number of spleen B-cells (CD45RA) is a rather sensitive parameter. In addition, reduced KLH-specific IgG is among the most sensitive parameters. Collectively, these findings suggest that the number of spleen mononuclear cells and KLH-specific IgG are the parameters that are affected after exposure to low doses of intravenous Ag-NP.

## Discussion

The present study is an extension of our previous intravenous 28-day repeated dose exposure study of 20 nm Ag-NP [14]. In the present study, and not in the previous one, the effect on the functional immune response, being the TDAR to KLH, was investigated. We confirm in the present study the observations of reduced body and thymus weight, increased spleen weight and spleen cell number, including increased percentages and numbers of the various spleen cell subsets measured. With the exception of the hemoglobin distribution width (HDW) which was decreased in our previous study, the exposure effects on red blood cell and hemoglobin parameters are similar. Most importantly, in the present study we show a reduced KLH-specific IgG response.

In our previous study [14], the most sensitive parameter was reduced thymus weight, with a BMD of 0.01 mg/kg (90% CI 0.001 – 0.14 mg/kg). In the present study a considerably higher BMD of 1.31 mg/kg (90% CI 0.76 - 3.03 mg/kg) was obtained. One explanation for this major difference is the fact that in the present study the animals were sacrificed 21 days after cessation of Ag NP exposure, whereas in our previous study the animals were euthanized 24 hours after final exposure. It should be mentioned that for all other parameters for which BMDs were determined in both studies, the difference in BMD between the two studies was much smaller, ranging between a 9-fold lower BMD to a 3-fold higher BMD.

Our study shows that the number of spleen mononuclear cells and KLH-specific IgG are the parameters that are affected after exposure to low doses of intravenous Ag-NP. The increased number of spleen mononuclear cells suggests immunostimulation, while reduced KLH-specific IgG suggests immunosuppression. Importantly, reduction of KLH-specific IgG suggests suppression of the functional immune system, leading to e.g. reduced resistance to infections by bacteria and viruses. As such, this observation of reduced KLH-specific IgG should be given more emphasis than an increased number of mononuclear cells in the spleen. Together with a similar KLH-specific IgM (and an increase at a higher dose), reduced KLH-specific IgG suggests that class switching from IgM to IgG is prevented by Ag-NP exposure. This effect is known to be induced by immunosuppressive drugs such as cyclosporine A and FK 506 [16]. The observation of reduced thymus weight, which is indicative for immune suppression by xenobiotics, is in line with reduced KLH-specific IgG and may suggest that impaired T-cell function is a cause for reduced KLH-specific IgG. Moreover, this finding underscores the importance to study TDAR to evaluate immunotoxicity and not to rely only on measuring immune cell subsets.

The present study was performed according to OECD guideline 407 (“Repeated dose 28-day oral study in rodents”) with some adjustments. First, animals were exposed to Ag-NP via intravenous administration. Second, the study was performed according to the BMD approach. Third, animals were immunized to KLH during exposure. Fourth, additional tests were performed to assess possible immunotoxic properties of Ag-NP. Fifth, only a limited number of organs was evaluated, i.e. mainly organs of the immune system, liver and blood.

The BMD approach has important advantages over determining the no-observed-adverse-effect-levels (NOAELs) and lowest-observed-adverse-effect-levels (LOAELs), being the more complete use of all individual data by the BMD approach and the fact that uncertainties about the value of a BMD can be quantified using statistical methodology. The advantage of dose-response modeling is that this uncertainty is made visible, whereas using the NOAEL it remains hidden [15].

An explicit statement on the benchmark response (BMR) is an improvement compared with the generally unknown response level associated with a NOAEL. However, it is often not clear what response level (BMR) can be considered as non-adverse and this choice needs consensus building [15]. Here we chose to use a BMR of 5% [17,18].

Wound dressings are the most likely way by which humans are exposed intravenously to silver nanoparticles. In patients with burns the median time to maximum silver levels was 9 days [19]; the median maximum silver level in serum was 56.8 μg/L. In rats 2 days after inflicting a deep partial-thickness wound and application of nano-crystalline chitosan wound dressing, a maximum blood silver level of 0.53 μg/g was measured [20]. This latter level is in the range of the lowest BMDL found in our study (Figure 1).

In our preceding study [14] 6 mg/kg bw was the (only) dose that showed some general toxicity as indicated by a reduction in body weight and was taken as the highest dose in the current study. A wide dose range was studied, between 8.2 μg/kg bw, corresponding to serum silver concentrations (57 μg/L) observed in humans after using nanosilver in wound dressings [19], and 6 mg/kg bw.

In a previous study by our group [21], the silver concentrations in blood after daily exposure to 0.1 mg/kg bw for 5 days was measured. Presence in organs was observed until day 17 after repeated exposure. Since in the present study the doses used are higher and the period of exposure is longer, it is likely that in the present study accumulation of the nanoparticles did occur.

While the observed effects are due to administration of nanosilver, it is very well possible that the mechanisms underlying the observed effects are (in part) caused by ionic silver. For instance, using an in vitro intestinal epithelium coculture model, the effects of the silver nanoparticles were likely exerted by the silver ions that are released from the nanoparticles [22].

A TDAR is dependent on both B-cell and T-helper cell functionality, and thereby also on the functionality of monocytes as antigen presenting cells, thus providing a read-out that evaluates the combined functionality of these immune cells [23]. Immunoglobulin class switching from IgM to IgG requires “help” from T-helper cells; therefore, KLH-specific IgG rather than KLH-specific IgM depends on the functionality of not only B-cells but also T-helper cells. In contrast to IgM, that depends only on B-cell activity, IgG requires “help” from T-cells and thereby also of antigen presenting cells. Next to being a functional parameter, another advantage of a TDAR is that it can be extrapolated from experimental animals to humans. Both its functional nature and its possibility for extrapolation are important for risk assessment. A TDAR that can be readily measured in humans is the vaccination titer, such as to measles and hepatitis B [24]. In the ICH S8 “Immunotoxicity studies for Human Pharmaceuticals”, an immune function study is recommended. Where a specific target is not identified, an immune function study such as the TDAR is recommended. Moreover, in the EPA Health Effects Test Guidelines “OPPTS 870.7800 Immunotoxicity” it is stated that hematology, lymphoid organ weights and histopathology alone are not sufficient to predict immunotoxicity. In conclusion, inclusion of a TDAR in immunotoxicity studies is of importance.

## Conclusions

The conclusion of the present study is that intravenous Ag-NP suppress the functional immune system. The possibility that the mechanisms underlying this effect are in fact caused by ionic silver after (slow) dissolution of the Ag-NP cannot be excluded.

## Methods

### Animals

Male Wistar derived WU (CRL:WI (WU)) rats, 8 weeks of age, were obtained from Charles River. Animals were bred under SPF conditions and barrier maintained during the experiment. Drinking water and conventional feed were provided ad libitum. Husbandry conditions were maintained according to all applicable provisions of the national laws, Experiments on Animals Decree and Experiments on Animals Act. The experiment was approved by an independent ethical committee (the Animal Experiments Committee of the National Institute for Public Health and the Environment) prior to the study.

### Chemicals

20 nm BioPure citrate Ag-NP in 2 mM citrate was obtained from NanoComposix, San Diego, CA, USA. The nanoparticle characteristics are presented in Table 2. The nanoparticles were provided sterile. The endotoxin level was tested using the gel clot method (LAL) and found to be < 10 IU/ml. Fatty acid tails were not detectable. The nanoparticle dispersions were freshly prepared every week.

**Table 2:** Characteristics of BioPure silver nanoparticles, as provided by the manufacturer

**Parameter**	
Size ± SD (nm)	21.0 ± 2.6
CV (%)	12.2
Size range (nm)	12.4 - 27.9
Concentration (mg/ml)	2
Number of particles (ml^-1^)	3.9 × 10^13^
Surface area per particle (nm^2^)	1.40 × 10^3^
Surface area (nm^2^/ml)	5.49 × 10^16^
Zeta potential (mV)	-40.8

### Experimental design

The animals were divided in 8 dose groups and were intravenously injected in the tail vein once daily for 28 days to either Ag-NP dispersion or vehicle control (phosphate buffer; PB). The daily doses Ag-NP were 0, 0.0082, 0.025, 0.074, 0.22, 0.67, 2, and 6 mg/kg bw.

In order to evaluate the effects of immunization, two additional groups were included in the study. These groups were not KLH-immunized, with one group receiving the highest Ag-NP dose and the other group not receiving Ag-NP.

Prior to and weekly during the experiment, the rats were weighed. Individual body weights of the rats were used to calculate the volumes to be administered. For each group, based on the weight of the heaviest animal of that group the dosing solution was prepared in such a way that the heaviest animal received 1 ml dosing solution, and the other animals in the group less than 1 ml. Dilution was in PB.

After euthanasia, body weights and the weights of liver, kidneys, spleen, and thymus were determined.

### Keyhole limpet hemocyanin immunization

**Figure 2:**
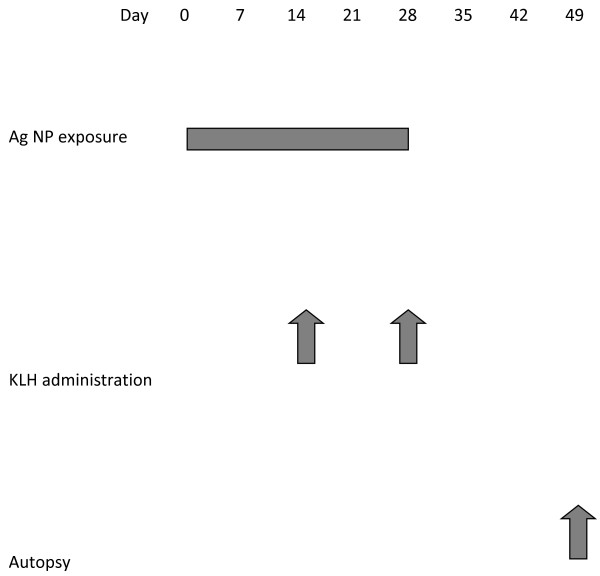
Experimental design.

KLH immunization was performed according to Tonk et al. [25]. Two hundred μl KLH (Pierce Biochemicals; 5 mg/ml in sterile water) was injected subcutaneously in the neck region. The primary and secondary immunizations were done at days 14 and 28, respectively, after starting the Ag-NP exposure (Figure 2). Serum was collected from blood samples obtained at day 49 and stored at -80°C until analysis for KLH-specific IgM and IgG.

### Hematology

Hematology was performed on blood samples obtained at autopsy. Blood was collected in EDTA-coated tubes. All hematology parameters were determined in an Advia 120 Hematology Analyzer (Siemens, Germany). These parameters were white blood cell (WBC) count, red blood cell (RBC) count, hemoglobin (HGB), hematocrit (HCT), mean corpuscular volume (MCV), mean corpuscular hemoglobin (MCH), mean corpuscular hemoglobin concentration (MCHC), red cell distribution width (RDW), hemoglobin distribution width (HDW), platelet (PLT) count, and mean platelet volume (MPV). In addition, the number and percentage of neutrophils, monocytes, lymphocytes, eosinophils, large unstained cells, basophils, and reticulocytes were measured. Next, blood smears were prepared for visual evaluation.

### Bone marrow

Cells were collected by flushing 4 ml Impuls Cytometer Fluid through the femur. The concentration of nucleated cells was determined in a Coulter Counter.

### Determination of spleen cell subsets

The protocol as described by Tonk et al. [25] was used. Spleen single-cell suspensions were examined for subset distribution by 3-color flow cytometry, using the following mAbs: allophycocyanin-conjugated mouse anti-rat CD3 (clone 1 F4; T-cells); R-Phycoerythrin–conjugated mouse anti-rat CD8a (clone OX-8; T-suppressor/cytotoxic cells); fluorescein isothiocyanate–conjugated mouse anti-rat CD4 (clone OX-35; T-helper cells); FITC-conjugated mouse anti-rat CD45RA (clone OX-33; B-cells); PE-conjugated mouse anti-rat CD161a (clone 10/78; NK cells); all from Pharmingen, San Diego, CA, USA. Single-cell suspensions were incubated with the conjugated mAbs for 30 min at 4°C in the dark. The cells were washed twice with wash buffer (5% bovine serum albumin (BSA; Sigma) in PBS), resuspended in 0.1% paraformaldehyde in PBS, and analyzed on a FACSCalibur flow cytometer (BD Biosciences, San Diego, CA, USA). A total of 10,000 events was recorded per sample and analyzed.

### Measurement of KLH-specific IgM and IgG

The protocol as described by Tonk et al. [25] was used. Serum samples were tested in an anti-KLH IgM or IgG specific ELISA using mouse monoclonal IgM (purified mouse anti-KLH IgM; BD Pharmingen) and rat monoclonal IgG2a (purified rat anti-KLH IgG2a, BD Pharmingen) for the preparation of standard curves. Flat-bottom plates (NUNC ImmunoPlate, Maxisorb) were coated overnight at 4°C with 5 μg/ml KLH (Pierce) in carbonate-bicarbonate buffer (0.05 M, pH 9.6; Sigma) and blocked with assay buffer (0.1% Tween-20 (Sigma-Aldrich) in PBS with 1% BSA) for 1 hour at room temperature (RT). The plates were washed with wash solution (0.05% Tween-20 in PBS), and diluted samples and standards were incubated for 45 min at 37°C. After washing, the plates were incubated with goat anti-rat IgM (Pierce) or IgG (Pierce), 1:5000 and 1:3000, respectively, for 30 min at 37°C. The plates were washed and tetramethylbenzidine substrate was added and incubated for 10 min in the dark at RT. The reaction was stopped with 10% (w/v) H_2_SO_4_. Optical density was measured at 490 nm using a Spectramax 190 spectrophotometer (Molecular Devices, Sunnyvale, CA, USA).

### Cell culture

The culture medium used was RPMI-1640 (Gibco, Grand Island, NY, USA) supplemented with 10% FCS, 100 μg/ml streptomycin, and 100 IU/ml penicillin. Cell suspensions were made by pressing the thymuses and spleens through a cell strainer (Falcon, Franklin Lakes, NJ, USA). Cells were counted using a Coulter Counter (Coulter Electronics, Luton, UK). Suspensions of thymocytes and splenocytes were cultured at 10^6^ cells/ml culture medium with 5 μg/ml Concanavalin A (MP Biomedicals, Irvine, CA, USA) in 96-well tissue culture plates (Nunc, Roskilde, Denmark) for 24 hours. Culture conditions were 37°C in a humidified atmosphere containing 5% carbon dioxide.

### Cytokine measurements

Multiplex panels measuring rat IFN-γ, IL-2, IL-10, IL-17, and TNF-α (thymocytes) and IL-1β, IL-2, IL-6, and IL-10 (splenocytes) were used. After incubation and washing steps [26], the beads were measured on a Bio-Plex (Bio-Rad).

### Statistical analysis of Ag-NP treatment

The dose-response data were analyzed using the BMD approach with PROAST software [27] (http://www.rivm.nl/proast). In this approach, a dose-response dataset is evaluated as a whole by fitting a dose-response model over the entire dose range studied.

Having fitted a dose-response model to the data, this curve is used to assess the BMD associated with the BMR of 5%. The choice of the model for deriving the BMD follows from a procedure of applying likelihood ratio tests to the five members of the following two nested families of models:

### Statistical analysis of KLH immunization

Statistical analysis of KLH immunization was performed using the independent-samples t-test (SPSS Inc., Chicago, IL, USA). Number of animals per group = 5.

## Abbreviations

Ag-NP: Silver nanoparticles; BM: Bone marrow cellularity; BMD: Benchmark dose; BMDL: Lowest 5% lower confidence bound; BMDU: Highest 95% upper confidence bound; BMR: Benchmark response; BSA: Bovine serum albumin; bw: Body weight; CI: Confidence interval; GI-tract: Gastro-intestinal tract; HCT: Hematocrit; HDW: Hemoglobin distribution width; HGB: Hemoglobin; KLH: Keyhole limpet hemocyanin; MCH: Mean corpuscular hemoglobin; MCHC: Mean corpuscular hemoglobin concentration; MCV: Mean corpuscular volume; MPV: Mean platelet volume; PLT: Platelet count; RBC: Red blood cell count; RDW: Red cell distribution width; RT: Room temperature; TDAR: T-cell dependent antibody response; WBC: White blood cell count.

## Competing interests

The authors declare that they have no competing interests.

## Authors’ contributions

RJV, ICMT, LTVDV, HVL, and WDJ were involved in conception and design of the study; LJDLFB, ERG and HWV were involved in acquisition of data; RJV, ICMT, HVL, and WDJ were involved in analysis and interpretation of data. RJV drafted the manuscript; HVL and WDJ critically revised the manuscript. All authors have given final approval of the version to be published.

## Supplementary Material

Additional file 1: Table S1 AgNP-exposure and KLH-immunization effects on body and organ weights. **Table S2.** AgNP-exposure and KLH-immunization effects on red blood cell parameters. **Table S3.** AgNP-exposure and KLH-immunization effects on white blood cell parameters. **Table S4.** AgNP-exposure and KLH-immunization effects on spleen cell subsets. **Table S5.** AgNP-exposure and KLH-immunization effects on KLH-specific IgG and IgM. **Table S6.** AgNP-exposure and KLH-immunization effects on cytokine production by thymus and spleen cells. Click here for file
