# Supplementary material for: Immunotoxicity of silver nanoparticles in an intravenous 28-day repeated-dose toxicity study in rats
Source: Part Fibre Toxicol. 2014 May 7;11:21. doi: 10.1186/1743-8977-11-21 (PMC4052812; doi:10.1186/1743-8977-11-21)
Supplement: Additional file 1: Table S1 — AgNP-exposure and KLH-immunization effects on body and organ weights. Table S2. AgNP-exposure and KLH-immunization effects on red blood cell parameters. Table S3. AgNP-exposure and KLH-immunization effects on white blood cell parameters. Table S4. AgNP-exposure and KLH-immunization effects on spleen cell subsets. Table S5. AgNP-exposure and KLH-immunization effects on KLH-specific IgG and IgM. Table S6. AgNP-exposure and KLH-immunization effects on cytokine production by thymus and spleen cells. [file 1743-8977-11-21-S1.docx]

| **treatment**  **(mg/kg bw)** | **body weight**  **(g)** | **liver weight**  **(g)** | **kidney weight**  **(g)** | **spleen weight**  **(mg)** | **thymus weight**  **(mg)** |
| --- | --- | --- | --- | --- | --- |
| KLH 0 | 372 ±16.0 | 12.6 ±0.81 | 2.88 ±0.21 | 550 ± 24 | 394 ± 45 |
| KLH 0.0082 | 394 ±13.1 | 13.3 ±0.62 | 2.95 ±0.21 | 553 ± 8 | 408 ± 45 |
| KLH 0.025 | 367 ±43.1 | 12.2 ±1.89 | 3.03 ±0.11 | 543 ± 49 | 386 ± 87 |
| KLH 0.074 | 397 ±47.9 | 12.8 ±0.59 | 2.85 ±0.13 | 538 ± 28 | 385 ± 71 |
| KLH 0.22 | 372 ±14.6 | 12.4 ±0.95 | 2.90 ±0.19 | 521 ± 34 | 363 ± 89 |
| KLH 0.67 | 389 ±17.4 | 12.9 ±0.66 | 3.01 ±0.17 | 549 ± 50 | 424 ± 60 |
| KLH 2 | 365 ±13.4 | 12.7 ±0.69 | 3.01 ±0.14 | 599 ± 41 | 355 ± 44 |
| KLH 6 | 341 ±12.6 | 11.7 ±0.78 | 2.76 ±0.21 | 755 ±139 | 312 ± 40 |
| CONT 0 | 379 ±19.8 | 12.4 ±0.77 | 2.93 ±0.21 | 532 ± 22 | 391 ±104 |
| CONT 6 | 350 ±23.0 | 12.5 ±1.28 | 2.81 ±0.26 | 795 ± 90 | 359 ± 57 |

**Table S1 AgNP-exposure and KLH-immunization effects on body and organ weights**

KLH, KLH-immunized; CONT, non-immunized. Mean ± SD (N=5, except KLH 0, where N = 10).

| **treatment**  **(mg/kg)** | **BM WBC**  **(x10^9^/l)** | **bl. WBC**  **(x10^9^/l)** | **RBC**  **(x10^12^/l)** | **Hb**  **(mmol/l)** | **Ht**  **(l/l)** | **MCV**  **(fl)** | **MCH**  **(fmol)** | **MCHC**  **(mmol/l)** | **RDW**  **(%)** | **HDW**  **(mmol/l)** | **PLT**  **(x10^9^/l)** | **MPV**  **(fl)** | **% ret** | **N ret**  **(x10^12^/l)** |
| --- | --- | --- | --- | --- | --- | --- | --- | --- | --- | --- | --- | --- | --- | --- |
| KLH 0 | 14.6 ± 7.1 | 6.7 ±0.93 | 8.7 ±0.37 | 8.8 ±0.42 | 0.42 ±0.019 | 48.4 ±0.6 | 1.01 ±0.01 | 20.9 ±0.2 | 12.7 ±0.2 | 1.49 ±0.06 | 647 ±54 | 7.90 ±0.77 | 1.33 ±0.19 | 99 ±26 |
| KLH 0.0082 | 13.0 ± 6.1 | 6.1 ±1.03 | 8.7 ±0.36 | 8.8 ±0.19 | 0.42 ±0.016 | 48.4 ±0.4 | 1.02 ±0.04 | 21.0 ±0.8 | 12.6 ±0.2 | 1.46 ±0.05 | 691 ±20 | 7.64 ±0.46 | 1.17 ±0.12 | 83 ±30 |
| KLH 0.025 | 19.6 ±11.9 | 7.6 ±1.65 | 8.9 ±0.39 | 8.8 ±0.52 | 0.43 ±0.022 | 48.2 ±0.7 | 1.00 ±0.03 | 20.6 ±0.3 | 12.6 ±0.1 | 1.45 ±0.06 | 713 ±65 | 7.38 ±0.52 | 1.25 ±0.23 | 111 ±21 |
| KLH 0.074 | 15.2 ± 7.5 | 6.4 ±1.07 | 8.7 ±0.14 | 8.7 ±0.08 | 0.42 ±0.008 | 48.7 ±0.6 | 1.00 ±0.00 | 20.6 ±0.2 | 12.6 ±0.2 | 1.46 ±0.05 | 679 ±51 | 7.28 ±0.52 | 1.25 ±0.06 | 107 ± 4 |
| KLH 0.22 | 16.8 ± 5.1 | 7.0 ±0.46 | 8.7 ±0.26 | 8.8 ±0.29 | 0.42 ±0.013 | 48.7 ±0.2 | 1.02 ±0.01 | 20.8 ±0.3 | 12.7 ±0.3 | 1.48 ±0.05 | 712 ±59 | 7.35 ±0.39 | 1.13 ±0.10 | 98 ± 7 |
| KLH 0.67 | 15.0 ± 5.6 | 6.7 ±1.30 | 8.7 ±0.28 | 8.7 ±0.28 | 0.42 ±0.015 | 48.9 ±0.6 | 1.01 ±0.01 | 20.7 ±0.3 | 12.8 ±0.2 | 1.48 ±0.04 | 645 ±68 | 7.50 ±0.43 | 1.28 ±0.10 | 112 ±10 |
| KLH 2 | 17.2 ± 4.3 | 5.9 ±1.60 | 9.3 ±0.33 | 8.7 ±0.30 | 0.43 ±0.013 | 46.0 ±0.5 | 0.94 ±0.00 | 20.4 ±0.2 | 13.5 ±0.2 | 1.56 ±0.05 | 732 ±17 | 7.18 ±0.65 | 1.31 ±0.14 | 123 ±14 |
| KLH 6 | 11.8 ± 6.4 | 7.1 ±1.12 | 9.6 ±0.27 | 8.6 ±0.26 | 0.43 ±0.011 | 44.6 ±0.4 | 0.89 ±0.01 | 19.9 ±0.2 | 14.4 ±0.1 | 1.60 ±0.07 | 627 ±106 | 8.30 ±1.24 | 1.60 ±0.18 | 119 ±35 |
| CONT 0 | 15.2 ± 5.7 | 6.1 ±0.86 | 8.8 ±0.30 | 8.7 ±0.26 | 0.42 ±0.013 | 47.7 ±0.4 | 0.99 ±0.01 | 20.8 ±0.1 | 12.7 ±0.2 | 1.53 ±0.05 | 707 ±52 | 6.73 ±0.21 | 1.24 ±0.09 | 95 ±23 |
| CONT 6 | 12.8 ± 6.5 | 6.0 ±0.98 | 9.2 ±0.32 | 8.4 ±0.14 | 0.41 ±0.011 | 44.9 ±0.5 | 0.91 ±0.03 | 20.3 ±0.6 | 14.6 ±0.1 | 1.60 ±0.07 | 647 ±100 | 8.92 ±2.37 | 1.64 ±0.16 | 134 ±35 |

**Table S2 AgNP-exposure and KLH-immunization effects on red blood cell parameters**

KLH, KLH-immunized; CONT, non-immunized. Mean ± SD (N=5, except KLH 0, where N = 10).

| **treatment**  **(mg/kg bw)** | **% neu** | **% lym** | **% mon** | **% eos** | **% luc** | **% bas** | **N neu**  **(x10^9^/l)** | **N lym (x10^9^/l)** | **N mon (x10^9^/l)** | **N eos (x10^9^/l)** | **N luc (x10^9^/l)** | **N bas (x10^9^/l)** |
| --- | --- | --- | --- | --- | --- | --- | --- | --- | --- | --- | --- | --- |
| KLH 0 | 17.4 ±5.6 | 79.3 ±6.1 | 1.65 ±0.34 | 0.55 ±0.17 | 0.91 ±0.42 | 0.29 ±0.18 | 1.18 ±0.49 | 5.3 ±0.64 | 0.11 ±0.036 | 0.04 ±0.013 | 0.06 ±0.026 | 0.02 ±0.016 |
| KLH 0.0082 | 16.6 ±3.4 | 80.1 ±3.6 | 1.62 ±0.23 | 0.71 ±0.19 | 0.56 ±0.21 | 0.15 ±0.11 | 1.01 ±0.27 | 4.8 ±0.80 | 0.10 ±0.016 | 0.04 ±0.018 | 0.05 ±0.043 | 0.01 ±0.010 |
| KLH 0.025 | 16.6 ±6.3 | 80.2 ±6.6 | 1.73 ±0.59 | 0.61 ±0.21 | 0.69 ±0.22 | 0.18 ±0.05 | 1.31 ±0.71 | 6.1 ±1.09 | 0.08 ±0.045 | 0.05 ±0.022 | 0.05 ±0.021 | 0.02 ±0.013 |
| KLH 0.074 | 17.2 ±2.4 | 80.0 ±2.5 | 1.62 ±0.28 | 0.59 ±0.14 | 0.55 ±0.14 | 0.15 ±0.05 | 1.08 ±0.14 | 5.1 ±0.97 | 0.10 ±0.025 | 0.04 ±0.007 | 0.03 ±0.017 | 0.01 ±0.008 |
| KLH 0.22 | 14.3 ±2.7 | 83.0 ±2.8 | 1.45 ±0.19 | 0.45 ±0.15 | 0.66 ±0.09 | 0.20 ±0.07 | 1.01 ±0.22 | 5.8 ±0.34 | 0.10 ±0.015 | 0.03 ±0.008 | 0.05 ±0.010 | 0.02 ±0.010 |
| KLH 0.67 | 18.0 ±3.9 | 78.9 ±4.2 | 1.52 ±0.40 | 0.59 ±0.13 | 0.84 ±0.27 | 0.17 ±0.06 | 1.22 ±0.43 | 5.3 ±0.92 | 0.10 ±0.054 | 0.04 ±0.011 | 0.06 ±0.021 | 0.01 ±0.007 |
| KLH 2 | 17.7 ±0.7 | 79.5 ±0.5 | 1.64 ±0.13 | 0.53 ±0.12 | 0.51 ±0.17 | 0.15 ±0.06 | 1.05 ±0.28 | 4.7 ±1.28 | 0.10 ±0.029 | 0.03 ±0.013 | 0.03 ±0.016 | 0.01 ±0.007 |
| KLH 6 | 20.9 ±4.5 | 74.5 ±4.3 | 2.56 ±0.19 | 0.70 ±0.26 | 1.28 ±0.41 | 0.19 ±0.04 | 1.46 ±0.36 | 5.3 ±0.95 | 0.18 ±0.040 | 0.05 ±0.017 | 0.09 ±0.042 | 0.02 ±0.005 |
| CONT 0 | 16.3 ±1.8 | 81.2 ±1.6 | 1.28 ±0.39 | 0.58 ±0.06 | 0.58 ±0.32 | 0.15 ±0.04 | 0.99 ±0.16 | 4.9 ±0.69 | 0.08 ±0.039 | 0.04 ±0.013 | 0.04 ±0.017 | 0.01 ±0.008 |
| CONT 6 | 19.3 ±4.1 | 76.5 ±3.8 | 2.72 ±0.45 | 0.59 ±0.19 | 0.90 ±0.29 | 0.12 ±0.04 | 1.14 ±0.20 | 4.6 ±0.93 | 0.16 ±0.025 | 0.03 ±0.008 | 0.05 ±0.018 | 0.00 ±0.005 |

**Table S3 AgNP-exposure and KLH-immunization effects on white blood cell parameters**

KLH, KLH-immunized; CONT, non-immunized. Mean ± SD (N=5, except KLH 0, where N = 10).

| **treatment**  **(mg/kg bw)** | **N spl**  **(x10^7^)** | **% CD8** | **%CD4** | **%CD3** | **%CD161** | **%CD45RA** | **CD4/**  **CD8** | **CD3/**  **CD45RA** | **N CD8**  **(x10^7^)** | **N CD4**  **(x10^7^)** | **N CD3**  **(x10^7^)** | **N CD161**  **(x10^7^)** | **N CD45RA**  **(x10^7^)** |
| --- | --- | --- | --- | --- | --- | --- | --- | --- | --- | --- | --- | --- | --- |
| KLH 0 | 49 ± 7.3 | 8.4 ±0.98 | 23.9 ±1.06 | 34.9 ±1.81 | 7.1 ±0.61 | 19.1 ±1.34 | 2.9 ±0.28 | 1.9 ±0.15 | 4.1 ±0.80 | 11.7 ±2.03 | 17.1 ±2.94 | 3.46 ±0.51 | 9.39 ±1.67 |
| KLH 0.0082 | 47 ± 4.0 | 7.8 ±1.71 | 22.5 ±0.58 | 32.9 ±1.60 | 7.1 ±0.72 | 19.3 ±0.99 | 3.0 ±0.66 | 1.7 ±0.13 | 3.7 ±0.97 | 10.5 ±1.02 | 15.5 ±1.79 | 3.33 ±0.40 | 9.06 ±0.93 |
| KLH 0.025 | 45 ± 5.0 | 7.9 ±1.07 | 23.6 ±0.89 | 34.2 ±1.47 | 7.1 ±0.86 | 17.7 ±1.70 | 3.0 ±0.37 | 2.0 ±0.17 | 3.6 ±0.62 | 10.7 ±1.30 | 15.5 ±1.92 | 3.18 ±0.43 | 8.08 ±1.51 |
| KLH 0.074 | 46 ± 4.9 | 8.0 ±0.83 | 24.8 ±1.73 | 35.3 ±1.93 | 7.3 ±0.43 | 18.0 ±2.51 | 3.1 ±0.36 | 2.0 ±0.31 | 3.7 ±0.47 | 11.4 ±1.24 | 16.3 ±1.58 | 3.38 ±0.48 | 8.39 ±1.93 |
| KLH 0.22 | 48 ± 6.2 | 8.2 ±0.92 | 24.1 ±1.05 | 35.0 ±1.34 | 6.1 ±1.54 | 17.6 ±1.63 | 3.0 ±0.41 | 2.1 ±0.22 | 3.8 ±0.69 | 11.1 ±1.24 | 16.2 ±1.89 | 2.87 ±0.85 | 8.17 ±1.52 |
| KLH 0.67 | 51 ± 2.8 | 8.2 ±1.28 | 22.4 ±1.10 | 32.9 ±1.23 | 6.9 ±0.62 | 19.2 ±1.27 | 2.8 ±0.47 | 1.8 ±0.14 | 4.1 ±0.69 | 11.3 ±0.73 | 16.7 ±1.02 | 3.46 ±0.21 | 9.70 ±0.86 |
| KLH 2 | 50 ± 1.5 | 8.8 ±1.43 | 24.8 ±1.15 | 36.3 ±0.82 | 8.7 ±0.92 | 18.2 ±2.46 | 2.9 ±0.68 | 2.1 ±0.25 | 4.3 ±0.66 | 12.3 ±0.73 | 18.0 ±0.57 | 4.27 ±0.35 | 8.97 ±0.98 |
| KLH 6 | 65 ±12.8 | 10.9 ±2.78 | 29.4 ±4.32 | 43.0 ±5.94 | 9.7 ±0.87 | 18.2 ±2.33 | 2.8 ±0.68 | 2.4 ±0.30 | 7.4 ±2.93 | 19.6 ±5.66 | 28.7 ±8.45 | 6.40 ±1.71 | 12.09 ±3.50 |
| CONT 0 | 51± 14.2 | 8.2 ±2.14 | 23.6 ±4.48 | 34.0 ±7.26 | 7.8 ±1.48 | 17.8 ±1.43 | 2.9 ±0.35 | 2.0 ±0.54 | 4.4 ±2.53 | 12.4 ±6.11 | 18.0 ±9.36 | 4.08 ±1.92 | 8.98 ±2.11 |
| CONT 6 | 67± 5.1 | 11.5 ±1.49 | 32.1 ±2.06 | 46.1 ±3.28 | 9.4 ±0.64 | 17.1 ±2.26 | 2.8 ±0.22 | 2.8 ±0.44 | 7.6 ±0.67 | 21.3 ±1.63 | 30.6 ±2.18 | 6.23 ±0.42 | 11.33 ±1.35 |

**Table S4 AgNP-exposure and KLH-immunization effects on spleen cell subsets**

KLH, KLH-immunized; CONT, non-immunized. Mean ± SD (N=5, except KLH 0, where N = 10).

| **treatment**  **(mg/kg bw)** | **KLH-IgG**  **log titer** | **KLH-IgM**  **log titer** |
| --- | --- | --- |
| KLH 0 | 6.45 ±0.38 | 4.51 ±0.23 |
| KLH 0.0082 | 6.35 ±0.36 | 4.37 ±0.36 |
| KLH 0.025 | 6.43 ±0.65 | 4.52 ±0.09 |
| KLH 0.074 | 6.38 ±0.47 | 4.75 ±0.23 |
| KLH 0.22 | 6.33 ±0.27 | 4.50 ±0.04 |
| KLH 0.67 | 6.07 ±0.37 | 4.51 ±0.20 |
| KLH 2 | 5.92 ±0.25 | 4.34 ±0.32 |
| KLH 6 | 6.23 ±0.57 | 5.03 ±0.52 |
| CONT 0 | 4.66 ±0.33 | 4.63 ±0.41 |
| CONT 6 | 4.71 ±0.20 | 4.46 ±0.23 |

**Table S5 AgNP-exposure and KLH-immunization effects on KLH-specific IgG and IgM**

KLH, KLH-immunized; CONT, non-immunized. Mean ± SD (N=5, except KLH 0, where N = 10).

| **treatment**  **(mg/kg bw)** | **thymus IFN-γ**  **(pg/ml)** | **thymus IL-2**  **(pg/ml)** | **thymus IL-10**  **(ng/ml)** | **thymus IL-17**  **(pg/ml)** | **thymus TNF-α**  **(pg/ml)** | **spleen IL-1β**  **(pg/ml)** | **spleen IL-2**  **(pg/ml)** | **spleen IL-6**  **(pg/ml)** | **spleen IL-10**  **(ng/ml)** |
| --- | --- | --- | --- | --- | --- | --- | --- | --- | --- |
| KLH 0 | 581 ±132 | 43.3 ± 7.8 | 7.18 ±2.00 | 1091 ±405 | 60.4 ± 8.9 | 26.1 ± 7.5 | 12.1 ±2.6 | 24.2 ± 9.1 | 2.72 ±0.52 |
| KLH 0.0082 | 445 ±102 | 48.2 ±10.2 | 6.75 ±1.22 | 1022 ±530 | 57.0 ± 9.0 | 26.5 ± 8.4 | 14.8 ±4.8 | 31.8 ±10.7 | 2.95 ±0.62 |
| KLH 0.025 | 528 ±233 | 44.9 ±14.3 | 5.87 ±1.41 | 990 ±428 | 56.5 ± 8.3 | 34.1 ±15.5 | 16.7 ±5.4 | 33.0 ±14.1 | 3.41 ±0.97 |
| KLH 0.074 | 629 ±137 | 47.0 ±11.0 | 7.96 ±2.10 | 1251 ±352 | 62.0 ± 9.0 | 29.6 ±11.8 | 12.1 ±2.8 | 30.0 ±18.2 | 2.85 ±0.64 |
| KLH 0.22 | 759 ±411 | 47.5 ±20.7 | 6.86 ±3.45 | 1003 ±620 | 69.7 ±19.2 | 40.2 ±19.9 | 17.6 ±8.0 | 56.8 ±38.3 | 3.47 ±1.43 |
| KLH 0.67 | 617 ±246 | 39.6 ±11.9 | 8.05 ±1.87 | 1218 ±221 | 62.0 ±11.1 | 28.0 ± 9.2 | 12.7 ±2.2 | 23.8 ±10.5 | 2.82 ±0.29 |
| KLH 2 | 484 ±157 | 49.1 ± 8.6 | 4.85 ±0.84 | 593 ±178 | 49.3 ± 7.5 | 56.8 ±40.8 | 25.4 ±9.2 | 79.0 ±76.2 | 3.46 ±1.05 |
| KLH 6 | 534 ±211 | 50.5 ±14.3 | 6.70 ±3.58 | 855 ±492 | 57.2 ±16.4 | 37.5 ±17.7 | 15.9 ±7.4 | 52.1 ±29.1 | 2.95 ±0.76 |
| CONT 0 | 580 ±163 | 37.8 ±10.3 | 7.13 ±1.55 | 883 ±229 | 57.0 ± 5.7 | 29.0 ± 9.0 | 12.0 ±7.4 | 33.9 ±22.8 | 2.61 ±0.89 |
| CONT 6 | 445 ±102 | 49.9 ± 7.5 | 4.94 ±1.07 | 557 ± 86 | 53.2 ± 5.5 | 58.8 ±35.8 | 20.5 ±9.0 | 55.2 ±31.6 | 3.01 ±0.73 |

**Table S6 AgNP-exposure and KLH-immunization effects on cytokine production by thymus and spleen cells**

KLH, KLH-immunized; CONT, non-immunized. Mean ± SD (N=5, except KLH 0, where N = 10).
